# Population-Attributable Risks for Ischemic Stroke in a Community in South Brazil: A Case-Control Study

**DOI:** 10.1371/journal.pone.0035680

**Published:** 2012-04-18

**Authors:** Adroaldo Baseggio Mallmann, Sandra Costa Fuchs, Miguel Gus, Flávio Danni Fuchs, Leila Beltrami Moreira

**Affiliations:** 1 Universidade de Passo Fundo- School of Medicine, Passo Fundo, Brazil; 2 Postgraduate Program in Cardiology; Universidade Federal do Rio Grande do Sul, Porto Alegre, Brazil; 3 Division of Cardiology, Hospital de Clinicas de Porto Alegre, Universidade Federal do Rio Grande do Sul, Porto Alegre, Brazil; Cardiff University, United Kingdom

## Abstract

**Background:**

Risk factors for ischemic stroke are mostly known, but it is still unclear in most countries, what are their combined population-attributable risk percent (PAR%). In a case-control study the individual odds ratios (ORs) and the individual and combined PAR%, including risk factors not addressed in previous studies were estimated.

**Methods:**

Cases and controls were selected from patients attending to an emergency department. Cases were patients aged with 45 years or more with the first episode of ischemic stroke, characterized by a focal neurological deficit or change in the mental status occurring during the previous 24 hours. Controls, matched to cases by age and gender, were selected from patients without neurological complaints.

**Results:**

133 cases and 272 controls were studied. Odds ratios for ischemic stroke were: atrial fibrillation (27.3; CI 95% 7.5–99.9), left ventricular hypertrophy (20.3; CI 95% 8.8–46.4), history of hypertension (11.2; CI 95% 5.4–23.3), physical inactivity (6.6; CI 95% 3.3–13.1), low levels of HDL-cholesterol (5.0; CI 95%2.8–8.9), heavy smoking (2.8; CI 95% 1.5–5.0), carotid bruit (2.5; CI 95% 1.3–4.6), diabetes (2.4; CI 95% 1.4–4.0) and alcohol abuse (2.1; CI 95% 1.1–4.0), The combination of these risk factors accounted for 98.9% (95% CI; 96.4%–99.7%) of the PAR% for all stroke.

**Conclusions:**

Nine risk factors, easily identified, explain almost 100% of the population attributable risk for ischemic stroke.

## Introduction

Stroke is a major health problem in many countries and it is the second leading cause of death worldwide [1], [2]. It is also the leading cause of work incapacity, and approximately 30–60% of all patients have some degree of physical disability after the acute event [3], [4]. In United States, it is estimated that there are 795 000 incident cases per year resulting in 134 000 deaths annually [5]. In Brazil, cerebrovascular disease is the main cause of death [6], [7] and the rates of mortality are considered the highest in the Americas, especially in the most deprived areas and black populations [8].

Despite overall declines in stroke and cardiovascular deaths in many countries, including the developed regions of Brazil [9], the stroke incidence is increasing in the United States [5]. On the other hand, a population-based study conducted in a restricted community of United Kingdom showed a reduction in stroke incidence from 1981 to 2004 [10]. The favorable trend was associated with improvement in the cardiovascular risk profile, and increased use of antiplatelet, lipid-lowering and anti-hypertensive treatments over time. Since approximately 70% of strokes are the first event, primary prevention is particularly important [5].

The INTERSTROKE case-control study, involving 22 countries worldwide showed that the combination of ten risk factors accounted for 90% population-attributable risk percent (PAR) for stroke [2] Not all potential risk factors [11], however, were investigated in the INTERSTROKE study. Moreover, only 151 from all 3000 cases were selected in South American countries.

The aim of this case-control study designed in a community of south Brazil was to estimate the combined PAR percent (PAR%) for the risk factors for ischemic stroke including modifiable risk factors that were not addressed individually in the INTERSTROKE study, such as carotid stenosis, atrial fibrillation and left ventricular hypertrophy.

## Materials and Methods

The sample was selected among consecutive patients admitted to the emergency department of São Vicente de Paulo Hospital, which is affiliated with the University of Passo Fundo, southern Brazil, between September 2009 and August 2010. Controls were selected at the same time, by frequency matching. Cases were patients with any focal neurological deficit or change in the mental status occurring during the previous 24 hours. Cerebral computed tomography (CT) scan was used to confirm the diagnosis of ischemic stroke. Individuals younger than 45 years of age and those with previous neurological disease were excluded.

Patients with CT findings of hemorrhage, tumor or hydrocephalus were also excluded. Controls were patients without neurological complains and with a normal score in the Glasgow coma scale. For every case, two age (±2 years) and gender frequency-matched controls were selected. All patients were managed according to usual routine care, and a neurologist confirmed the diagnosis of stroke. A written informed consent was obtained from the patients or from next-of-kin if necessary. The study was approved by the Ethics Committee of our Institution. Demographic and clinical baseline data were collected using standardized interview and physical examination.

Hypertension was defined by a previous medical diagnosis of hypertension or use of blood pressure-lowering drugs. Physical activity was assessed using a questionnaire adapted from the *The Northern Manhattan Stroke Study*. The questionnaire recorded the frequency and duration of 19 different physical activities during the last year A series of yes/no responses were recorded for each of the questions, posed as “In the last year, have you engaged in physical activity?” Each affirmative response was followed by two other questions: “On average, how many times did you perform this activity each week?” and “On average, how many minutes each time?” and “How many months in the year?” From these responses the frequency and duration of each activity were computed [12]. Physical inactivity was defined as the lack of any regular exercise during leisure time. Fasting blood samples were collected to measure total cholesterol, HDLcholesterol (HDL-C) and glucose. LDL-cholesterol (LDL-C) was calculated using Friedewald formula [13]. The cutoff point for LDL-C was 160 mg/dl and for HDL-C the respective values for men and women were 50 mg/dl and 40 mg/dl [14]. Fasting glucose greater than 126 mg/dl was used to diagnose diabetes [15]. Patients who smoked 20 cigarettes a day were considered to be a current heavy smoker. The usual daily intake of alcohol in a typical week over the previous 6 months was determined through the quantity–frequency method, based on the kind of beverage consumed. Men consuming 30 g of ethanol or more per day and women consuming 15 g of ethanol or more per day were classified as abusers [16]. The presence of carotid bruit was assessed during the clinical evaluation in the emergency department. Measured or informed weight and height were used to calculate the body mass index (BMI). BMI greater or equal than 30 kg/m2 was used to define obesity. Electrocardiograms (ECG) were done in all patients, and the presence of atrial fibrillation and left ventricular hypertrophy were considered for analysis. The voltage criteria of Sokolow and Lyon was used to define left ventricular hypertrophy [17].

The sample size of 405 patients had a power of 80% to estimate the odds ratio (OR) of 2.0 for hypertension, smoking and alcohol abuse between cases and controls. The respective values for diabetes, physical inactivity and atrial fibrillation were 1.7, 1.6 and 1.6. The differences between means were compared using Student's t-test. Chi-square test was used to compare proportions. The crude and adjusted odds ratios (OR) and 95% confidence intervals (CI) were calculated through conditional logistic regression analysis.

Adjusted odds ratios (ORs) for risk factors were derived from their respective coefficients in the multivariate logistic regression models. In these models the corresponding odds ratio was adjusted for smoking status, hypertension and diabetes. Statistical analyses were conducted with Statistical Package for Social Sciences (SPSS®, version 16, Il, USA). The PAR% for each risk factor was estimated from the matched case-control study, controlling for confounding factors, as well as the combined PAR%, using the software Interactive Risk-Attributable Program (IRAP, USA National Cancer Institute, 2002).

## Results

In total, 405 patients were selected, including 133 cases and 272 controls. Three additional stroke cases had missing data and were not included in the analysis. Characteristic of cases and controls patients are showed in Table 1. In face of matching, age and gender distribution were similar among cases and controls. All risk factors were more common among cases

**Table 1:** Characteristics of case and control patients.

	Cases (N = 133)	Controls (N = 272)	P
Sex			
Male	85 (63.9%)	175 (64.3%)	0.93
Caucasians	123 (92.5%)	238 (87.5%)	0.13
Age (years)			
45–55	19 (14.3%)	32 (11.8%)	0.86
56–65	55 (41.4%)	109 (40.1%)	
66–75	39 (29.3%)	86 (31.6%)	
>75	20 (15.0%)	45 (16.5%)	
Years of school			
≤8 years	87 (65.4%)	144 (52.9%)	0.003
9–11 years	39 (29.3%)	83 (30.5%)	
>11 years	7 (5.3%)	45 (6.5%)	
BMI≥30 Kg/m^2^	73 (54.9%)	191 (70.2%)	0.002
Smoking status			
Heavy smoker	38 (28.6%)	64 (23.5%)	<0.001
Alcohol abuse	36 (29.3%)	37 (15.9%)	0.005
Physical inactivity	121 (91%)	160 (58.8%)	<0.001
History of Hypertension	76 (58.9%)	76 (28.3%)	<0.001
Carotid bruit	32 (24.1%)	27 (9.9%)	0.01
Diabetes	45 (33.8%)	50 (18.4%)	0.001
Atrial fibrillation	19 (14.3%)	4 (1.5%)	<0.001
Left ventricular hypertrophy	75 (56.4%)	22 (8.1%)	<0.001
Total cholesterol (mg/dl)	227.3±65.2	210.8±44.5	0.009
HDL-C (mg/dl)	39.40±8.7	44.60±9.4	<0.001
LDL-C (mg/dl)	156.9±49.2	140.8±36.4	0.001

**Figure 1:**
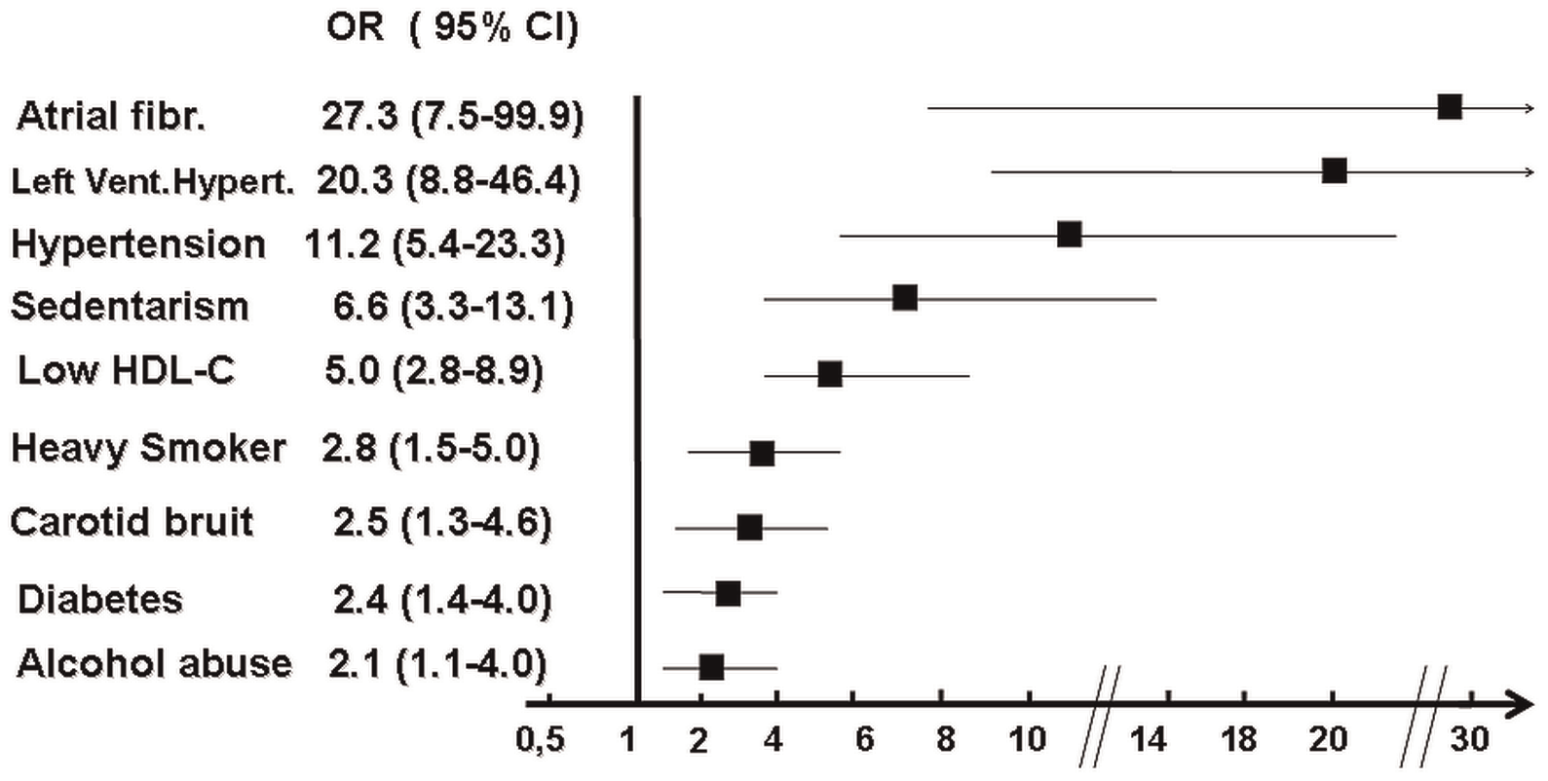
Odds ratios (95% CI) for ischemic stroke: results of a logistic regression adjusting for hypertension, diabetes and smoking status.

Figure 1 shows the OR (95%CI) for logistic regression models for each risk factor, adjusted for hypertension, diabetes and smoking status. Atrial fibrillation, left ventricular hypertrophy and hypertension were the strongest risk factors associated with ischemic stroke, with ORs exceeding 10. Obesity and high LDL-C were not independently associated with ischemic stroke.

Considering only the risk factors significantly associated with ischemic stroke in the logistic regression models, the PAR% was calculated for each risk factor. Table 2 shows that hypertension, physical inactivity, low HDL-C and left ventricular hypertrophy had the highest PAR% values. The overall PAR% was calculated including all the risk factors shown in Table 2. The combination of hypertension, atrial fibrillation, left ventricular hypertrophy, presence of carotid bruit, heavy smoking status, diabetes, alcohol abuse, HDL-C and physical inactivity explained 98.9% (95% CI: 96.4%–99.7%) of the ischemic stroke incidence.

**Table 2:** PAR% for each risk factor for ischemic stroke.

Risk Factor	RAP %	CI 95%
History of Hypertension	84.9	72.5–92.3
Physical inactivity	77.2	61.1–87.9
Left Ventricular. Hypertrophy	53.6	44.4–62.6
Diabetes	19.5	10.4–33.5
Heavy smoker	19.2	10.7–31.9
Carotid bruit	14.3	7.1–26.5
Alcohol abuse	14.1	5.8–30.4
Atrial fibrillation	13.8	8.8–20.9
HDL-Cholesterol	57.1	43.7–70.0

## Discussion

The present case-control study confirmed that traditional risk factors for ischemic stroke explain most of its incidence. The combination of hypertension, low levels of HDL-C, atrial fibrillation, left ventricular hypertrophy, the presence of carotid bruit, heavy smoking status, diabetes, alcohol abuse and physical inactivity accounted for 99.0% of the stroke cases. This finding means that we could achieve 99% reduction in stroke risk by the strict control of these modifiable risk factors. Differently from other studies [2], [18], obesity was not independently associated with ischemic stroke, probably in face of the association between obesity and history of hypertension, which had the highest PAR% value. Moreover, BMI has been not considered the best measurement of excess of adiposity [19].

Cohort studies have investigated mainly the contribution of lifestyle indicators (smoking, body mass index, physical activity, healthy diet and alcohol consumption) for the incidence of stroke incidence, identifying that they explain approximately 50% of PAR% of ischemic stroke. These estimates are within our findings, since we included these risk factors and others, such as high blood pressure, atrial fibrillation and left ventricular hypertrophy [20], [21]. In guidelines for the primary prevention of stroke [5], risk factors are classified according to their potential for modification (nonmodifiable, modifiable, or potentially modifiable) and strength of evidence (well-documented, less well documented). Moreover, the strength of the association is shown in terms of relative risk and PAR% for most risk factors. Among the risk factors presented as well-documented and modifiable risk factors from the current guidelines, sickle cell disease, postmenopausal hormonal therapy, the nutritional status and the use of oral contraceptives were not analyzed in our study. The combined PAR% of all risk factors was not informed in the current guidelines, but, in our analyses, the inclusion of sickle cell disease, post-menopause hormonal therapy, nutritional status and the use of oral contraceptives would minimally modified the overall PAR% estimate, since it was already close to 100%. As in our study, the INTERSTROKE [2] and the Prospective Studies Collaboration meta-analysis of cohort studies [22] showed that increased concentration of total cholesterol was not associated with risk of ischemic stroke, whereas increased concentration of ApoA1 and ApoB was associated with a reduced and increased risk for ischemic stroke, respectively. We did not measure the apolipoproteins following the current recommendations of the guidelines for assessment of the cardiovascular risk profile in asymptomatic adults [23]. Depression and psychosocial stress that were evaluated in the INTERSTROKE study are not listed in the current guidelines as well-documented and modifiable risk factors for stroke [5]. On the other hand, we investigated the risks of atrial fibrillation, left ventricular hypertrophy and carotid bruit. A combination of nine easily assessed risk factors explained almost 100% of the case condition, in comparison with 90% in the INTERSTROKE study [2].

Although the main evidences for the overall PAR% of risk factors for ischemic stroke came from the results of the INTERSTROKE case-control study, its external validity was questioned. Thrift et. al pointed out that that people living in rural disadvantaged settings could have other risk factors that could be more relevant to the assessment of the risk profile for ischemic stroke [11]. In our study we adopted a methodological approach including universal risk factors that can be easily assessed when a stoke diagnosis was addressed. Moreover, differently from the INTERSTROKE study, we specifically measured the ORs of atrial fibrillation, left ventricular hypertrophy and carotid bruit, which are well recognized risk factors directly associated with stroke incidence.

Some potential limitations and strengths of our study deserve mention. First, there is the possibility of recall bias typical of case-control studies. The data collection by certified research assistants, using a standardized instrument, and checking of some questionnaires with the next-of-kin may have minimized the risk for recall bias. Second, case-control design is more prone to bias than prospective studies and therefore has lower hierarchy to establish the evidence. For example, blood pressure was not measured before the occurrence of stroke, and the diagnosis of hypertension may have been missed in some patients, leading to a potential underestimation of the contribution of hypertension. On the other hand, the feasibility of this design allows to investigate risk factors for stroke in a scenario with more limited resources [24]. Third, the absence of confirmation of carotid stenosis by an image method is a potential limitation of our study, since carotid bruits are poor predictors of either underlying carotid stenosis or stroke risk in asymptomatic patients. On the other side, Pickett et al. described in a meta-analysis with 28 cohort studies that patients with a carotid bruit have four times the risk of transient ischemic attack and twice the risk of stroke when compared with controls [25]. If the negative likelihood ratio described by Johansson et al. is applied to our high risk sample (cases), where the prevalence of carotid bruit was 24%, the absence of this clinical sign would have a post-test probability of only 5% for severe carotid stenosis [26] Fourth, our patients were recruited from a single center and patients with less than 45 years and with previous neurological disease were not selected. Therefore extrapolation of our findings to other populations should be cautious. And finally, in face of the design of data collection, we could not explore the association between risk factors and subtypes of ischemic strokes, such as embolic, thrombotic, and of small vessels. The control group employed in our study is one of its strengths, since cases and controls were originated in the same population.

In conclusion, nine risk factors, easily identified by history, physical examination and ordinary lab examinations, explain almost 100% of the population attributable risk for ischemic stroke.
